# A Case Report of Sweet Syndrome and Hemorrhagic Bullae Associated With Acute Myeloid Leukemia With Myelodysplasia-Related Changes

**DOI:** 10.7759/cureus.90523

**Published:** 2025-08-19

**Authors:** Tanachanan Kukongviriyapan, Walairat Sitthikornsawat

**Affiliations:** 1 Internal Medicine, Samut Sakhon Hospital, Ministry of Public Health Thailand, Affiliation to Siriraj Hospital, Samut Sakhon, THA; 2 Dermatology, Samut Sakhon Hospital, Ministry of Public Health Thailand, Affiliation to Siriraj hospital, Mahidol University, Samut Sakhon, THA

**Keywords:** acute myeloid leukemia (aml), aml with myelodysplasia-related changes (aml-mrc), hemorrhagic bullae, myelodysplasia-related changes (mrc), sweet syndrome (ss)

## Abstract

Sweet syndrome is a rare condition characterized as a febrile neutrophilic dermatosis. We present an atypical presentation of Sweet syndrome in female patient in her late 80s, characterized by acute fever, concurrent pancytopenia, and a unique constellation of cutaneous findings consisting of painful papulonodular lesions and hemorrhagic bullae with compatible histopathology, underscoring the necessity for clinicians to consider acute myeloid leukemia with myelodysplasia-related changes as a potential comorbidity in Sweet syndrome, particularly in cases with comparable clinical features. This report is particularly significant as it demonstrates the rapid clinical deterioration and emergence of complications, culminating in an unfavorable outcome despite early therapeutic intervention. The infrequent reporting of this unique presentation highlights the need for further exploration of its diagnostic and management relevance in patients with Sweet syndrome who exhibit hemorrhagic bullae manifestations.

## Introduction

Sweet syndrome (SS), historically known as acute febrile neutrophilic dermatosis, is an inflammatory dermatosis characterized by the abrupt onset of fever, elevated neutrophil counts, painful erythematous skin lesions, and a distinctive dermal infiltrate of mature polymorphonuclear cells [1-2]. While its precise pathogenesis remains incompletely understood, it is hypothesized to involve a complex interplay of genetic predisposition and environmental triggers [3].

This report presents an exceptional case of bullous SS with hemorrhagic features in an 89-year-old female with acute myeloid leukemia with myelodysplasia-related changes (AML-MRC), an association documented only sporadically in the literature [2]. The patient’s rapid clinical decline, culminating in fatal sepsis despite early intervention, highlights the prognostic challenges in malignancy-associated SS, particularly in immunocompromised hosts.

This case highlights three key points: diagnostic complexity, as bullous SS may mimic other diseases and requires meticulous clinicopathological correlation; therapeutic dilemma, where balancing immunosuppressive treatment against infection risks in AML-associated neutropenia poses management challenges; and rarity, since hemorrhagic bullous SS with AML-MRC is an underreported entity with poorly defined outcomes [2]. We aim to enhance recognition of SS as a paraneoplastic phenomenon in AML-MRC and emphasize the importance of multidisciplinary vigilance in immunocompromised patients.

## Case presentation

An 89-year-old Thai female with a medical history of essential hypertension and chronic kidney disease presented with complaints of fatigue, dizziness, and anorexia lasting one week. On the third day of hospitalization, the patient developed a fever. Empirical broad-spectrum antibiotic therapy, consisting of intravenous piperacillin-tazobactam and azithromycin, was initiated; however, there was no discernible clinical improvement. Cutaneous manifestations were observed at the time of the initial hospital presentation, occurring prior to the commencement of antibiotic treatment.

**Figure 1:**
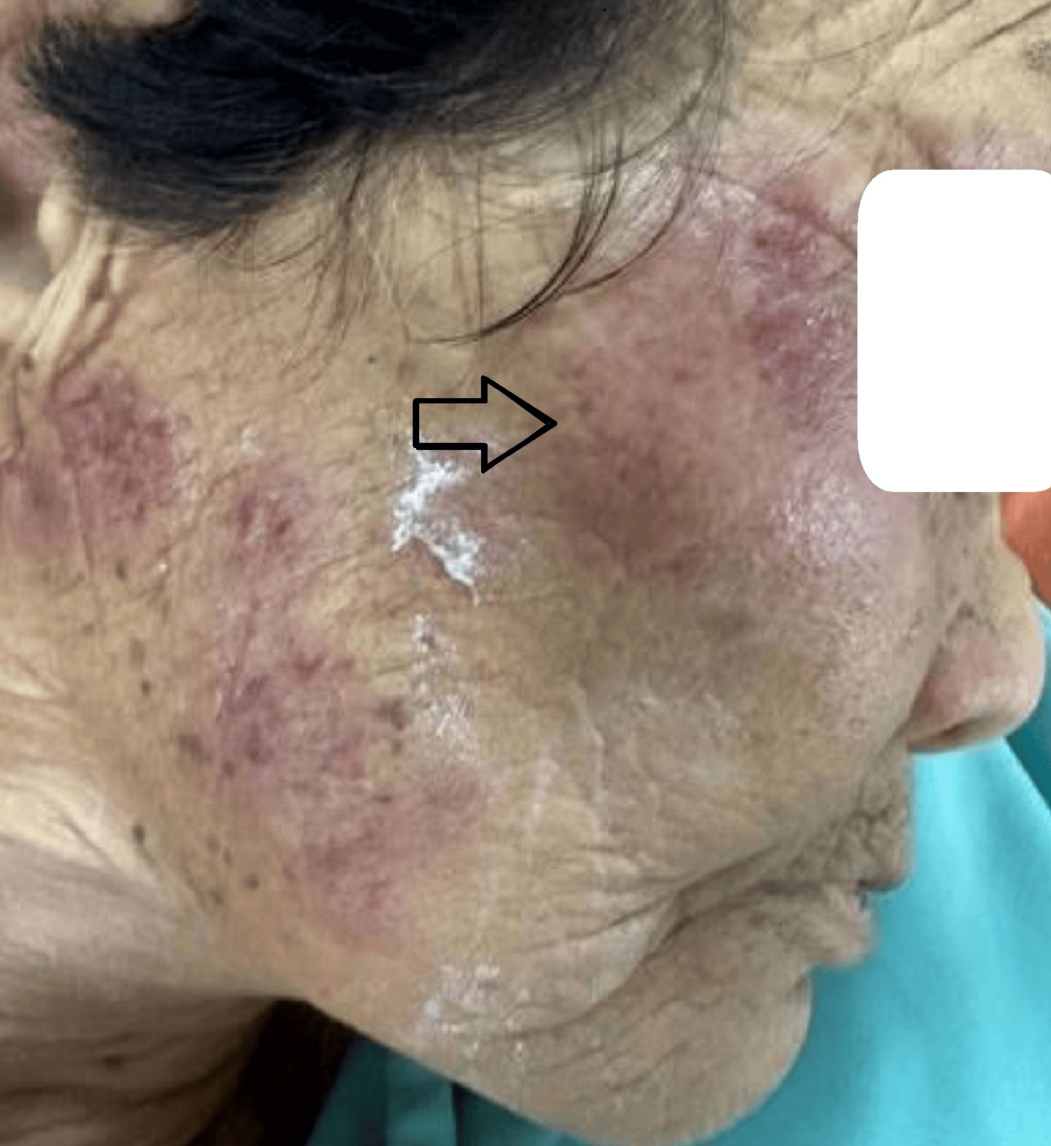
Multiple ill-defined non-blanchable erythematous to violaceous papules, nodules, and plaques on the right jaw and right cheek

**Figure 2:**
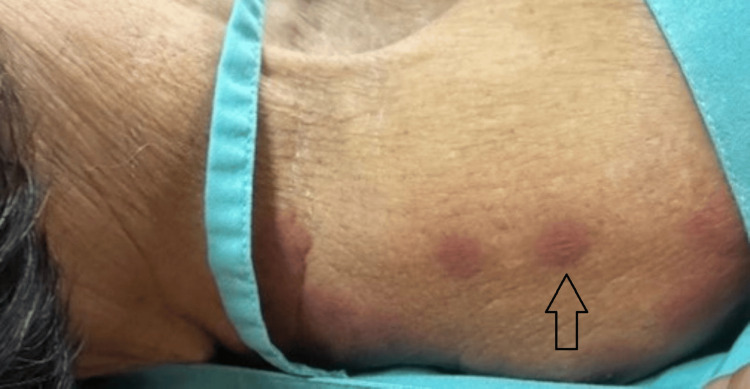
Multiple well-circumscribed erythematous edematous nodules on the right arm

**Figure 3:**
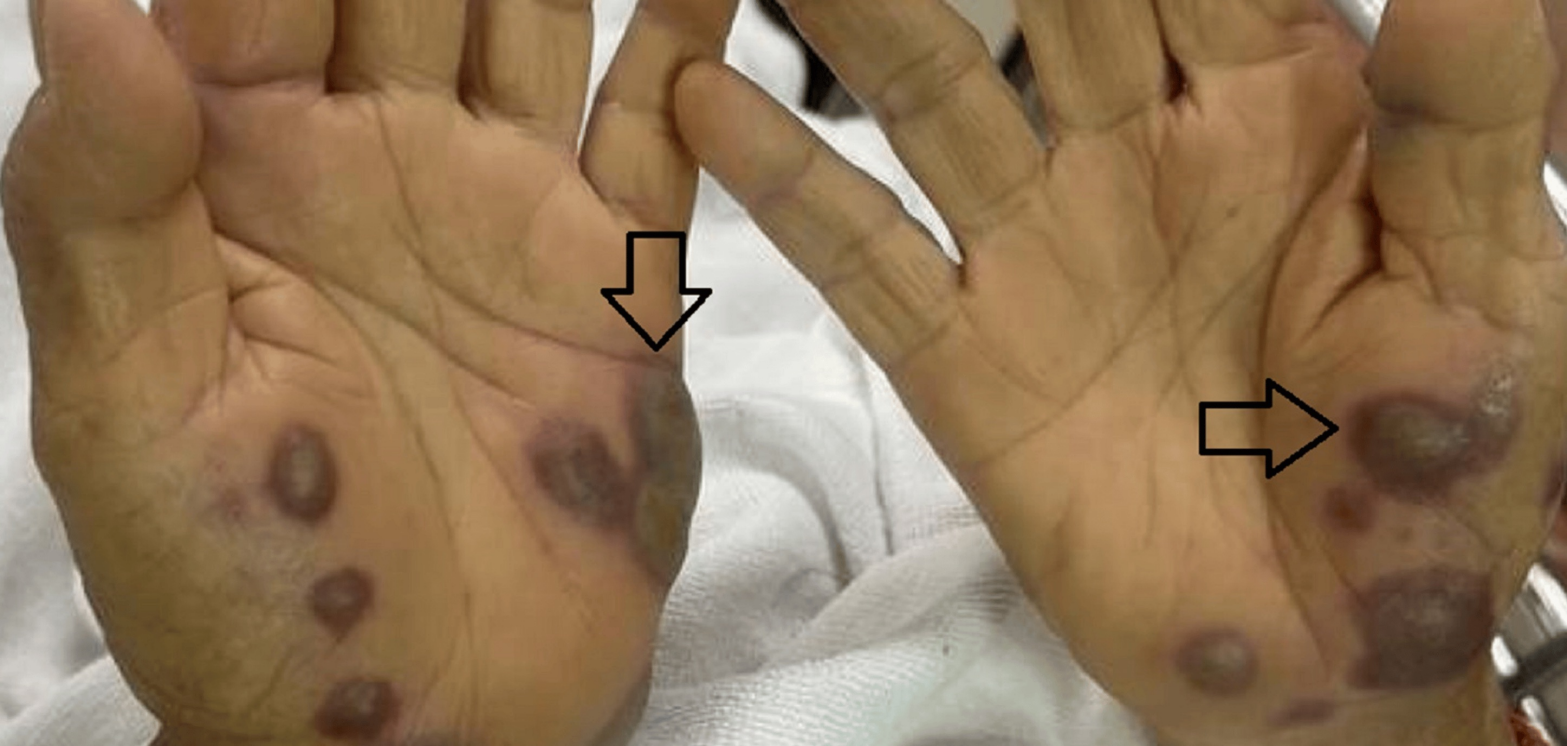
Multiple well-circumscribed hemorrhagic bullae on pseudovesicle plaques on both palms (Especially on the right palm)

**Figure 4:**
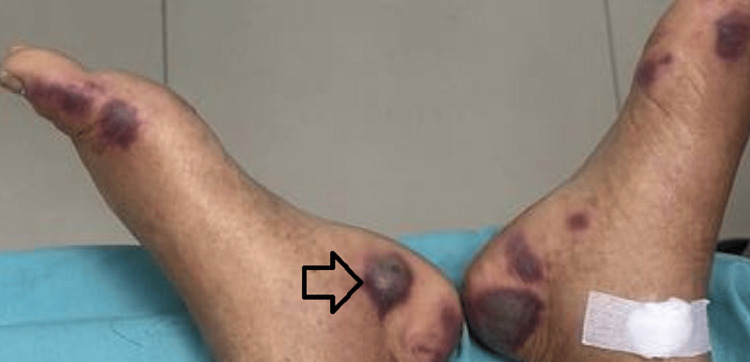
Multiple well-circumscribed hemorrhagic bullae on erythematous bases on both soles

Examination revealed multiple discrete, ill-defined, non-blanchable, erythematous to violaceous, painful papules, nodules, and plaques localized to the face, neck, and upper extremities (Figures 1-2). Additionally, hemorrhagic bullae on pseudovesicular plaques were observed on the palms and soles (Figures 3-4). A subsequent examination demonstrated pale conjunctivae and the absence of mucocutaneous lesions, hepatosplenomegaly, or lymphadenopathy. The cardiovascular, respiratory, and gastrointestinal systems were unremarkable. The patient had been on long-term folic acid and amlodipine therapy prior to admission. She reported no prior adverse drug reactions and no pre-hospitalization medication adjustments.

A clinical review revealed an acute-onset skin rash accompanied by abrupt-onset fever, prompting a differential diagnosis that prioritized infectious etiologies, SS, and drug reactions. In light of the patient’s advanced age and clinical findings suggestive of pancytopenia, hematologic malignancies, including acute leukemia and cutaneous T-cell lymphoma, were considered highly probable. Considering the overall context, the case favored malignancy-associated over drug-induced SS. The differential diagnosis further encompassed inflammatory or autoimmune conditions, specifically bullous SS, bullous leukocytoclastic vasculitis, bullous pemphigoid, and pemphigus vulgaris. Additionally, infectious etiologies, including meningococcemia, should be considered, especially in elderly or immunocompromised patients presenting with hemorrhagic blebs and unstable vital signs.

However, microbiological cultures remained crucial for exclusionary purposes. Given the inability to differentiate the lesion based solely on clinical presentation, a skin biopsy was performed on the right arm (Figure 2).

**Figure 5:**
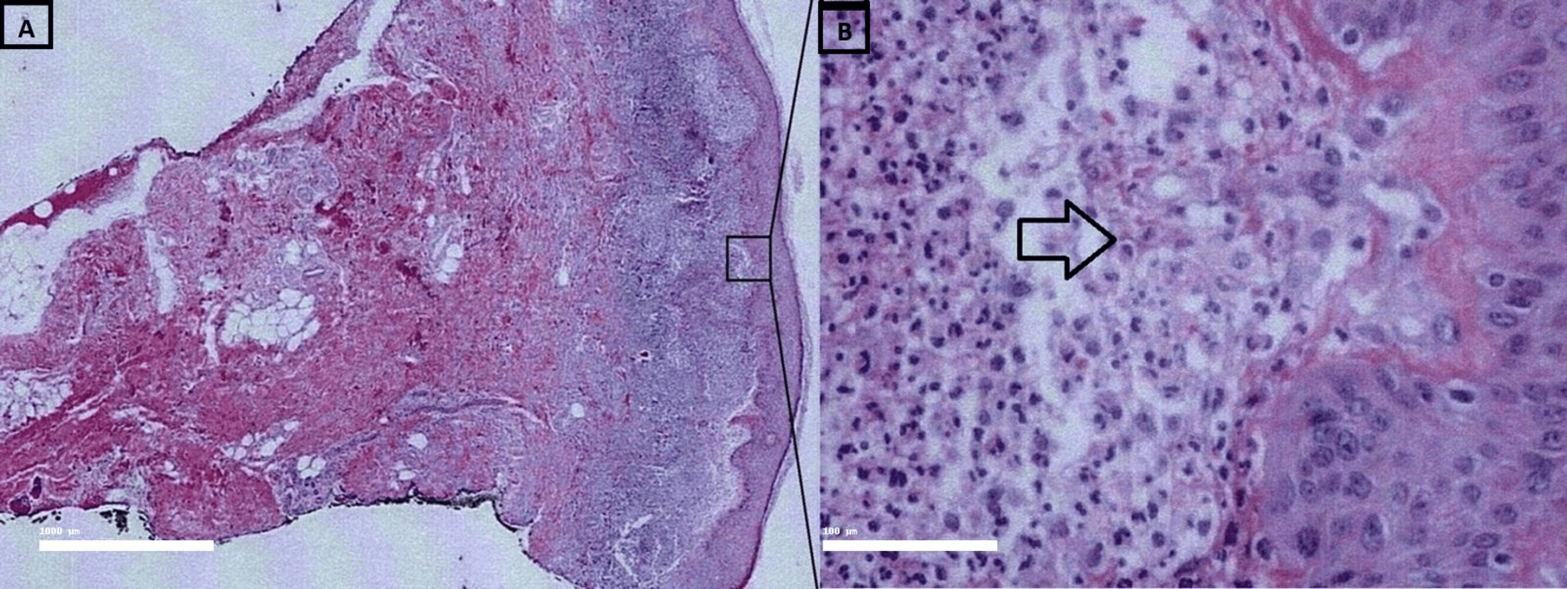
A) Acanthotic epidermis with hyperkeratosis and focal parakeratosis, accompanied by superficial dermal edema (H&E stain, ×40), B) Dense neutrophilic inflammatory infiltrate in upper dermis with focal subepidermal separation (H&E stain, ×400) The formalin-fixed specimen consists of a punch biopsy of skin, measuring 0.4×0.4×0.5 cm. (A) Lower-power view (original magnification, ×40) of a skin biopsy showing an acanthotic epidermis with hyperkeratosis and focal parakeratosis along with superficial dermal edema with dense cellular infiltrate in papillary and upper reticular dermis. (B) High-power view (original magnification, ×400) highlighting dense inflammatory infiltrate of predominantly neutrophils with some lymphocytes in the upper dermis with focal subepidermal separation, but without evidence of leukocytoclastic vasculitis.

Histopathological examination of the affected skin revealed acanthotic epidermis with hyperkeratosis and focal parakeratosis along with superficial dermal edema with dense cellular infiltrate in papillary and upper reticular dermis. The dermis exhibited superficial dermal edema, with infiltration of neutrophils and lymphocytes, focal subepidermal separation, no signs of vasculitis, no acantholysis or eosinophilic infiltrate as seen in bullous pemphigoid, and no leukemic blasts or granulocytic differentiation indicative of leukemic cutis. These features are suggestive of SS (Figure 5).

Hematological analysis revealed pancytopenia, elevated C-reactive protein levels, and abnormal hepatic transaminases (Table 1). Microbiological cultures from blood and skin samples yielded negative results. Wright’s and Tzanck-stained smears of skin lesions demonstrated no evidence of acantholytic cells or infectious organisms.

**Table 1:** Blood test results from the patient’s first presentation The data in the table demonstrate several hematological alterations alongside elevated C-reactive protein (CRP) levels. Specifically, the data indicate anemia, leukopenia (with a notable neutrophilic predominance), thrombocytopenia, and elevated hepatic transaminases.

Tests	Reference Ranges	Blood Levels Baseline 3 Months Prior to Admission	Blood Levels at Initial Presentation	Blood Levels at 1.5 Months after the Initial Presentation
CRP (mg/L)	0.5-5.0	-	20	25
Hemoglobin (g/dL)	13.0-17.0	11.2	7.7	5.8
White blood cell (×10^9^/L)	4.0-10.0	3.8	2.3	1.3
Platelet count (×10^9^/L)	150-450	110	30	31
Neutrophils (%)	40-80	55	67.8	34
Lymphocytes (%)	20-40	20	27.2	59
AST(U/L)	0-50	26	54	60
ALT (U/L)	0-50	30	62	68

Furthermore, screening for autoimmune antibodies, encompassing antinuclear antibody (ANA), anti-dsDNA, anti-desmoglein-1 IgG, anti-desmoglein-3 IgG, and anti-basement membrane IgG, demonstrated negative findings.

Flow cytometric analysis and bone marrow examination confirmed AML-MRC, characterized by 42% blasts expressing CD13, human leukocyte antigen (HLA)-DR, myeloperoxidase (MPO), megakaryocytic dysplasia, and left shift in maturation. Chromosomal analysis identified a complex karyotype, including deletions of 5q and 7 (Table 2). No evidence of malignant lymphoma was detected.

**Table 2:** Immunophenotyping characterization and chromosome karyotype CD: cluster of differentiation; HLA-DR: human leukocyte antigen-DR; MPO: myeloperoxidase 1. Flow cytometry of peripheral blood revealed 42% myeloid blasts expressing CD13, CD33, MPO, CD34, and HLA-DR, consistent with acute myeloid leukemia. 2. Bone marrow biopsy showed dysplastic megakaryocytes and left-shifted maturation, suggestive of a myelodysplastic component. 3. Flow cytometry of bone marrow biopsy revealed an immature (blast) cell population expressing CD5, CD34, and CD38, highly suggestive of acute leukemia, particularly AML, given the co-expression of CD13 and CD33 found in peripheral blood. 4. Chromosomal analysis identified a complex karyotype with deletions of 5q and 20q, monosomy 7 and 18, supporting the diagnosis of AML with myelodysplasia-related changes (AML-MRC).

Site	Investigation	Results (Immunohistochemistry/Cell Type /Karyotype)	
Peripheral blood	Flow cytometry	CD13+, CD33+, CD34+, CD117+, HLA-DR+, MPO+ blasts 42%	
Bone marrow	Bone marrow pathology	Moderate hypercellular trilineage marrow	
		Megakaryocytic dysplasia	
		Left shifting of erythroid cells	
	Flow cytometry	Numerous degenerated cells and a mixed population of reactive T and B lymphocytes	CD5+, CD38+, CD34+, CD20-, Kappa-, Lambda-
	Chromosome study	Four cells showed female pattern with deletion of chromosome 5,20, and loss of one chromosome 7,18	44,XX,del(5)(q13q33),-7,-18del(20)(q11,2)(4)/46,XX,del(5) (q13q33)(13)/46,XX(3)

Treatment consisted of colchicine (1.8 mg/day) and high-potency topical corticosteroids. Colchicine was chosen instead of corticosteroids (first-line for SS) due to concerns about infection risk associated with systemic corticosteroids in AML, especially given the patient’s immunocompromised state. Supportive care for anemia was administered with oxymetholone (50 mg/day) and blood transfusions. In light of the patient’s advanced age, compromised functional status, and limited medical coverage, neither intensive chemotherapy nor hypomethylating agents for AML-MRC were administered.

One month after treatment, the patient experienced fatigue, bone pain, and decreased appetite, which correlated with laboratory findings indicative of anemia. She subsequently received blood transfusions and analgesics. Due to the clinical improvement of the SS rash, the colchicine dosage was reduced to 1.2 mg daily. Approximately 1.5 months following the initiation of treatment, the patient returned with clinical manifestations of severe sepsis and febrile neutropenia, culminating in rapid mortality.

## Discussion

SS is described as an acute febrile neutrophilic dermatosis [1]. SS exhibits a female predominance, with a reported male-to-female ratio of 1:4, and frequently manifests between the third and sixth decades of life [4].

SS is classified into three subtypes: classical, malignancy-associated, and drug-induced SS. These subtypes share a common clinical context, characterized by the sudden onset of tender erythematous nodules, papules, or plaques, with a predilection for the face, neck, and upper extremities. These lesions typically exhibit an asymmetrical distribution accompanied by systemic manifestations [1,5,6].

Malignancy-associated SS have revealed distinctive characteristics, particularly bullous or ulcerated presentations [2]. The symptoms of SS may present concurrently with, precede, or follow the emergence of the associated malignancy [3]. Hematologic malignancies, particularly AML, are the most pertinent. Other hematologic associations include lymphoma and myelodysplastic syndrome (MDS) [5].

Drug-induced SS was considered unlikely. Amlodipine, the patient’s long-term medication, showed no temporal relationship with symptom onset, which typically occurs within six weeks, and no supporting evidence exists in the literature or reputable case reports linking it to SS. Reported drug associations include granulocyte-colony stimulating factor (G-CSF), azathioprine, and all-trans retinoic acid (ATRA), none of which had been used in this patient [7,8].

Cutaneous variants of SS encompass bullous, cellulitis-like, necrotizing, neutrophilic dermatosis of the dorsal hands, and generalized pustular SS [6]. Bullous SS, a rare variant, presents with hemorrhagic bullae or flaccid or tense blisters on acral surfaces, extremities, trunk, and face, often accompanied by dermoepidermal junction separation [6]. Bullous SS has been reported with AML and both active and inactive ulcerative colitis [6,9].

Extracutaneous manifestations of SS have been reported across multiple organ systems, including pulmonary, hepatobiliary, and hematologic systems [3].

In the pathogenesis of AML-MRC-associated SS, dysregulated marrow or leukemic blasts release excessive pro-inflammatory cytokines, particularly IL-1β, IL-6, G-CSF, GM-CSF, and TNF-α, into the circulation, driving neutrophil activation, chemotaxis, and cutaneous infiltration as part of a paraneoplastic hypersensitivity reaction. The resulting cytokine storm sustains neutrophil recruitment in the skin, producing the characteristic lesions [4,8-10].

The diagnostic criteria for SS require both major criteria and at least two minor criteria (Table 3) [3]. This case met the two major criteria of abrupt onset of painful skin lesions and compatible histopathology, as well as two minor criteria, namely fever and concurrent hematologic malignancy.

**Table 3:** Diagnostic criteria for SS SS: Sweet syndrome The diagnostic criteria for SS require both major criteria and at least two minor criteria.

Major Diagnostic Criteria	Minor Diagnostic Criteria
1. Sudden appearance of painful erythematous papules, nodules, or plaques	1. Fever (>38°C)
	2. Association with one of the following: antecedent upper respiratory or gastrointestinal infection, vaccination, malignancies, inflammatory disorders, or pregnancy
2. Histopathological profile indicative of a dense dermal neutrophilic infiltrate without evidence of leukocytoclastic vasculitis	3. Significant response to systemic corticosteroids or potassium iodide
	4. Supporting laboratory findings consist of erythrocyte sedimentation rate >20 mm/h, leukocytosis (WBC > 8 × 10^9^/L), neutrophilia (>70%), elevated C-reactive protein (at least three of four)

Histopathological examination reveals papillary dermal edema and a dense, band-like infiltrate of neutrophils within the dermis with no evidence of vasculitis [1].

Therapeutic interventions focus on addressing the underlying malignancy. Additionally, systemic corticosteroids remain the first-line treatment, administered at a dosage of 1 mg/kg/day, either orally or intravenously, for a duration of 3 to 4 weeks [3].

Steroid-sparing therapeutic options include colchicine (1.5 mg/day) and potassium iodide (900 mg/day) [3,11]. Second-line therapeutic agents, including cyclosporine, dapsone, and indomethacin [3]. Refractory cases necessitate high-dose methylprednisolone, and biologic agents have shown promise, including anti-TNFα agents (infliximab), anti-IL-1 agents (anakinra), and anti-IL-6 agents (tocilizumab) [6,12].

In a retrospective analysis of 83 patients with SS by Nelson et al. (2018), 44% were associated with malignancy, with AML accounting for 29% of total cases. Vesiculobullous lesions were more frequently observed among patients with malignancy (25%) [9].

A retrospective study of 37 SS cases by Zheng et al. (2020) reported that 10 cases were associated with malignancy. Among these, nine cases involved hematologic malignancies, with AML and MDS each accounting for 44% (4 cases each). Furthermore, the study emphasized investigating hematologic malignancies in patients exhibiting anemia and thrombocytopenia [13].

A retrospective analysis conducted by Gil-Lianes et al. (2023) involving 93 patients diagnosed with SS. Malignancy was identified in 37.7% of patients with papules or plaques, 14.3% of those with vesiculobullous lesions, and 28.6% of patients with nodules. Notably, no malignancies were observed in patients with pustular lesions [14].

Across three retrospective cohorts totaling over 200 patients, the association between SS and hematologic malignancies, particularly AML, was consistently observed. While Nelson et al. reported 29% AML prevalence, Zheng et al. found AML in 44% of malignancy-associated SS cases. Gil-Lianes et al. further highlighted the lesion-type correlation, with vesiculobullous lesions associated with malignancy in 14.3% of patients [9,13-14].

## Conclusions

This case underscores AML-MRC as a key consideration in elderly patients with bullous SS and pancytopenia. The subsequent severe disease progression, characterized by febrile neutropenia and the development of severe infections in an immunocompromised patient, emphasizes the pivotal role of interprofessional collaboration in the timely diagnosis and management of the therapeutic challenges encountered in this clinical context.

Despite the implementation of symptom-directed therapy, including steroid-sparing strategies, targeted treatment of comorbidities, meticulous wound care, and rigorous infection control, the patient’s prognosis was driven by AML-MRC progression. Therapeutic options were limited due to the patient’s frailty and AML complexity, underscoring the challenges in treating malignancy-associated SS. Future studies should explore whether early AML-directed therapy optimizes outcomes in similar clinically challenging cases.
